# IQOS point-of-sale marketing: a comparison between Arab and Jewish neighborhoods in Israel

**DOI:** 10.21203/rs.3.rs-3953025/v1

**Published:** 2024-03-01

**Authors:** Amal Khayat, Hagai Levine, Carla J Berg, Lorien C Abroms, Zongshuan Duan, Yan Wang, Cassidy R LoParco, Daniel Elbaz, Yuxian Cui, Yael Bar-Zeev

**Affiliations:** Hebrew University of Jerusalem Braun School of Public Health and Community Medicine; Hebrew University of Jerusalem Braun School of Public Health and Community Medicine; George Washington University School of Public Health and Health Services: The George Washington University Milken Institute of Public Health; George Washington University School of Public Health and Health Services: The George Washington University Milken Institute of Public Health; Georgia State University School of Public Health; George Washington University School of Public Health and Health Services: The George Washington University Milken Institute of Public Health; George Washington University School of Public Health and Health Services: The George Washington University Milken Institute of Public Health; Hebrew University of Jerusalem Faculty of Medicine; George Washington University School of Public Health and Health Services: The George Washington University Milken Institute of Public Health; Hebrew University of Jerusalem Braun School of Public Health and Community Medicine

**Keywords:** IQOS, heated tobacco products, point-of-sale, marketing

## Abstract

**Background:**

Philip Morris International’s (PMI) IQOS, with its heatsticks (HEETS), is the heated tobacco product with the largest global market share. IQOS and/or electronic cigarettes use rate is higher among Arabs vs. Jews in Israel. This paper aims to compare IQOS point-of-sale (POS) marketing strategies, and regulatory compliance in Arab vs. Jewish neighborhoods in Israel.

**Methods:**

We integrated data from two separate studies including a cross-sectional survey with IQOS retailers (December 2020-April 2021) and audits of POS that sold IQOS/HEETS (April 2021-July 2021) in 5 large cities in Israel, after marketing restrictions including a POS display ban and plain packaging became effective in Israel (January 2020). The survey included 69 POS (21 Arab, 48 Jewish neighborhoods) and the audits included 129 POS (48 Arab, 81 Jewish neighborhoods). Comparisons of IQOS marketing strategies between POS in Arab and Jewish neighborhoods were conducted using Chi-Square test, Fisher’s exact test or Mann-Whitney test, as appropriate. Thematic analysis was used to analyze open-ended questions.

**Results:**

Most marketing strategies, such as promotions to customers, were uniform across POS in Arab and Jewish neighborhoods. The most noteworthy differences were that a higher proportion of retailers from Arab neighborhoods were invited to IQOS parties (47.6% vs. 21.7%, p<0.05) and reported personal communication with a PMI representative (80.0% vs. 51.2%, p<0.05). Additionally, PMI representatives assisted POS in both Arab and Jewish neighborhoods in implementing the display ban by providing free compliant cabinets and product placement instructions, and directly interacted with customers. POS in Arab neighborhoods were more compliant with the display ban (25.5% vs. 8.8%, p<0.05), but less compliant with plain packaging (62.5% vs. 79.3%, p<0.05).

**Conclusions:**

There were not many notable differences in IQOS marketing across POS in Arab vs. Jewish neighborhoods, but PMI utilized marketing elements of cultural significance, especially for POS in Arab neighborhoods, such as more personal communication and invitation to social events. Continuous surveillance of tobacco POS marketing and legislation compliance is needed, with a special focus on demographic/location-based differences.

## Background

IQOS is a heated tobacco product (HTP), composed of an electronic device that heats tobacco sticks called HEETS, and is manufactured by Philip Morris International (PMI). IQOS was first launched in Japan in 2014, and is currently sold in more than 70 countries, dominating the HTP global market.^[1, 2]^ The point-of-sale (POS) environment is one of the most important but underregulated venues for tobacco marketing.^[3–5]^ Marketing materials and price promotions at POS increase brand awareness and impulse purchasing,^[5–8]^ attract susceptible populations (mainly youth), and serve as smoking cues that might hinder quit attempts and trigger relapse.^[5, 7–9]^ In addition, tobacco products are usually placed at the eye-level, behind the counter, or in a place visible for most, if not all, customers.^[10–13]^ Some tobacco companies pay for dedicated counter spaces or supply the POS with free display cases.^[10]^

Studies have shown that POS display and advertisement bans are effective tobacco control measures that can support quit attempts and lead to reduced smoking rates over time.^[4, 9, 14–16]^ POS marketing might be especially important for new products like IQOS. The way a new product like IQOS is promoted to retailers at POS, and their attitude towards it, might affect their direct-to-consumer approach.^[5]^

There is evidence to suggest that tobacco companies might use distinctive marketing strategies to differently target POS in specific areas, for example; in neighborhoods representing greater proportions of specific ethnic groups.^[17]^ A 2015 systematic review revealed PMI’s use of an “Integrated Retail Demographic Database Micro-Marketing Tool”, which utilized data gathered from demographic census and retail pricing to customize campaigns and offerings, including price promotions, directed at specific POS in certain areas.^[18]^ Several studies have found more tobacco marketing and lower cigarette prices in neighborhoods with lower socioeconomic status (SES) and large proportion of ethnic minority individuals;^[19–22]^ however, other studies have documented no clear associations.^[23, 24]^ In the United States (US), menthol cigarettes marketing was more prevalent in neighborhoods with large proportions of African American residents, while smokeless tobacco was mainly advertised in predominantly White neighborhoods.^[20]^

Israel is a predominantly Jewish country, with the Arab population being its largest ethnic minority group (21.1% of the total population).^[25, 26]^ More than 80.0% of Arabs reside in all or majority-Arab localities, 95.0% of which are of low SES.^[27]^ Tobacco and nicotine use is higher among Arabs than Jews (24.4% vs. 19.1% for cigarette; 2.8% vs. 1.2% for IQOS and/or electronic cigarette).^[28]^ IQOS first entered the Israeli market in 2016 and has been regulated the same as all other tobacco and nicotine products since 2017.^[25, 26, 29]^ Among other measures, this includes an advertisement ban in TV, radio, digital media and POS (2019), a POS display ban where all tobacco and nicotine products should be concealed at all times (2020), and plain packaging requirements for all of these products (2020).^[25, 26, 29]^

In a previous Israel-based study,^[3]^ concealed POS audits were carried out at 80 POS in 2019 and 2020 to assess marketing materials and regulatory compliance before the POS display ban and plain packaging went into effect (January 8 2020);^[3]^ IQOS/HEETS marketing materials and price promotions were uncommon, but IQOS/HEETS special displays were found at 20% of the audited POS.^[3]^ However, this study only included a small sample of POS in Arab neighborhoods (n = 5), and therefore could not compare whether marketing strategies differed between POS in predominantly Arab vs. Jewish neighborhoods.

This study aims to assess and compare IQOS marketing strategies to and at POS, regulatory compliance and retailers’ attitudes towards IQOS between POS in Arab vs. Jewish neighborhoods in Israel.

## Methods

### Study design

This manuscript integrates data from two sources:

1. Cross-sectional survey among retailers at POS that ever-sold IQOS/HEETS (December 2020-April 2021).
2. Concealed POS audits of retailers that were currently selling IQOS/HEETS (April 2021-July 2021).

For each data collection, POS in Jewish neighborhoods were randomly selected from the IQOS Israel website, and all POS in Arab POS were included in the sample (but data from the survey and audits were not matched).^[5]^

#### Cross-Sectional Survey

##### Settings and procedures

This study examined IQOS marketing strategies at POS in Israel via a phone survey with owners or managers of POS that ever sold IQOS/HEETS in five large cities in Israel: Haifa, Jerusalem, Tel Aviv (all have both Jewish and Arab populations), Beer Sheva (a predominantly Jewish city), and Nazareth (a predominantly Arab city) in December 2020 to April 2021.^[5]^ The initial sample included the total of 713 POS (n= 86 in Arab neighborhoods and n=627 in Jewish neighborhoods) across these cities, which were then called by phone to participate in the survey. This approach yielded an insufficient number of POS in Arab neighborhoods (n=5) to conduct any comparisons (n=38 POS in Jewish neighborhoods). Thus, additional recruitment efforts included attempting in-person survey data collection by research assistants (RAs) in a matched sample (by city and SES) of POS in Arab and Jewish neighborhoods that could not be reached by phone.

Of the overall n=314 POS successfully contacted/visited (n=76 in Arab neighborhoods, n=238 in Jewish neighborhoods), surveys were completed in a final sample of n=69 (n=21 in Arab neighborhoods, n=48 in Jewish neighborhoods). Those that were contacted/visited but did not result in completed either: 1) never sold IQOS/HEETS (n=82 overall; n=16 in Arab and n=66 in Jewish neighborhoods); 2) did not consent (n=163 overall; n=40 in Arab and n=124 in Jewish neighborhoods). Retailers (either POS manager or owner) who completed the survey were compensated with a 100 New Israeli Shekel (NIS) online voucher.

##### Data collection tool

The survey assessed: 1) POS characteristics (i.e., type of store, belonging to a chain, neighborhood SES); 2) participant characteristics (i.e., age, sex, job position at the location, cigarette use status, IQOS use status); 3) number of HEETS flavors sold [<4, 4–5, or all 6 HEETS flavors], and presence of a special display for IQOS/HEETS; 4) marketing strategies directed at the POS (e.g., free HEETS samples for the retailer’s personal use, incentives for sales) with an option to freely elaborate on each item; 5) marketing strategies directed at the customers including promotions (e.g., free HEETS samples for the customers, price discounts) with an option to elaborate on each item, and advertisements (i.e., if the retailer ever promoted IQOS/HEETS online, via social media, via print media, or inside the POS, recoded into yes/no for each and later recategorized to “any form of ads”); 6) a question assessing how retailers would communicate with customers about IQOS and/or HEETS (*“How would you describe the IQOS/HEETS to your customers who might ask about your tobacco products or IQOS?”*) with 9 separate check boxes (e.g., *“IQOS is less harmful compared to traditional combustible cigarettes”*); 7) an additional open-ended question asking them to describe their personal attitudes of IQOS/HEETS; 8) interactions with a PMI salesperson (the manufacturer of IQOS) such as providing direction on product placement, target market, how to communicate with customers, and providing information about IQOS/HEETS in comparison to other tobacco products (recoded into yes/no for each and with an additional variable of “any interaction”); and 9) PMI’s reactions to the new tobacco legislation (i.e., retailers were provided education on the new legislation; free cabinets, etc.).

#### POS audits

##### Settings and procedures

From April to July 2021, trained research assistants conducted concealed, in-person POS audits among POS that currently sold IQOS/HEETS in the same large cities (Beer Sheva, Haifa, Jerusalem, and Tel Aviv, and Nazareth). The final total sample was 129 POS (48 Arab and 81 Jewish neighborhoods). Audits were conducted using a validated surveillance tool, developed based on the Standardized Tobacco Assessment for Retail Settings (STARS), and adapted for IQOS^[3]^

### Data collection tools

The audit tool assessed: 1) POS characteristics (e.g., type of store, belonging to a chain, neighborhood SES); 2) marketing materials inside or outside the POS such as IQOS/HEETS special display, QR code, or signage for products sold (coded as “any ad” if marketing materials were found for any product); 3) price promotions; 4) visibility of the sold products (sold and visible, sold and not visible, or not sold); 5) prices (i.e., least expensive price of a cigarette pack and HEETS) and any price promotions across products; 6) placement (i.e., within 30 cm of toys or candy, and/or within one meter of the floor); and 7) regulatory compliance (e.g., presence of minimum age signage, presence of a “no smoking” sign, all tobacco and nicotine products in plain packaging, and all tobacco and nicotine products are completely covered and not visible; if a product was visible the POS was coded as noncompliant).

### Data analysis

Descriptive analysis was conducted using counts and percentages (%) for categorical variables and mean (SD) for continuous variables (e.g., age, product price). Bivariate analyses were conducted using Chi-Square test, Fisher’s exact test or Mann-Whitney test, with Bonferroni correction as appropriate, to assess the differences between POS in Arab and Jewish neighborhoods. For all analyses, SPSS v27 was used, and a p<0.05 was considered statistically significant. Open coding and thematic analysis were used by the lead researcher (AK) to code and analyze free-text and open-ended questions, which were validated by a senior researcher (YBZ).

## Results

### Cross-Sectional Survey

#### POS and participant (manager/owner) characteristics

Table 1 summarizes the POS and participant (manager/owner) characteristics from the retailers’ survey, overall and comparing retailers at Arab vs. Jewish neighborhoods. The surveyed POS were mainly grocery stores (37.7%, n=26) or convenience stores not within a gas station (34.8%, n=24). A higher proportion of POS in Arab neighborhoods were grocery stores (71.4% vs. 22.9%, *p=0.001*), and all of them (100.0%) were located in low- and medium-SES neighborhoods, compared to 64.6% of POS in Jewish neighborhoods (*p=0.009*).

#### IQOS marketing strategies

Table 2 summarizes IQOS/HEETS marketing strategies overall, and compares retailers in Arab vs. Jewish neighborhoods. Compared to Jewish neighborhoods, a higher proportion in Arab neighborhoods carried less than 4 HEETS flavors (66.7% vs. 17.4%, *p<0.001*) and less IQOS special displays (25.0% vs. 53.2%, *p=0.034*) (Table 2).

More retailers in Arab vs. Jewish neighborhoods received invitations to IQOS events/parties (47.6% vs. 21.7%, *p=0.032*) and paraphernalia (30.0% vs. 2.2%, *p=0.002*). The most prevalent form of promotions targeting customers were price discounts (18.8% and 44.4% of POS in Arab and Jewish neighborhoods, respectively, p=0.069) (Table 2). Only retailers in Arab neighborhoods mentioned receiving lighter stands (n=2), flights abroad (n=1), swimming competitions (n=1), and receiving points when referring customers (n=1). More than half of all POS carried IQOS promotional materials (Table 2); these were mainly electronic and/or non-electronic signs that said “here you can buy heated tobacco products” or “heated tobacco units”, small flags that say the same or advertise a price promotion, and/or special display cases for IQOS/HEETS. A few retailers mentioned that PMI sent saleswomen to set up a small stand and promote IQOS directly to customers (6 in Arab neighborhoods and 3 in Jewish neighborhoods).

#### Open-ended questions

Retailers from POS in Arab neighborhoods emphasized their part in promoting the product to their customers and connecting the customers with sales representatives. For example, one participant used her personal experience as a promotional strategy: *“When people come to my shop and see me use it they get curious and start asking me about it, I tell them about my personal experience and how I used to smoke Marlboro but when I switched to IQOS I stopped coughing in the morning and it doesn’t stink your clothes or furniture”*.

Another participant stated that the POS was acting as a “middle man” by connecting the customer with a PMI sales representative: *“the shop was the intermediary; the company’s representative asked us to connect him with the customers if anyone asks about IQOS or was interested in trying it”*, and mentioned collecting ID numbers and phone numbers to register customers for a user database, and received points for each person. Others referred to the representative’s direct interaction with customers; *“the representative and I try to tell customers about IQOS, that it can meet their requirements and is less harmful and smoke-free”*.

#### Retailers’ attitudes towards IQOS and interactions with PMI representatives

**Figure 1:**
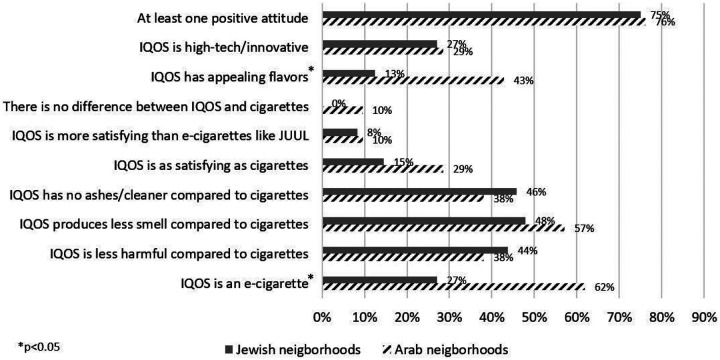
Retailers’ attitudes towards IQOS: this shows the percentage of retailers who chose each statement (not mutually conclusive) about how they would describe the IQOS/HEETS to their customers who might ask about their tobacco products or IQOS. The start sign denotes statistically significant differences, using Chi-square test (p<0.05)

More retailers from POS in Arab neighborhoods stated that IQOS is an e-cigarette (61.9% vs. 27.1%, *p=0.006*) and found its flavors to be appealing (42.9% vs. 12.5%, *p=0.009*) (figure 1: retailers’ attitudes towards IQOS). Overall, 42.0% of retailers stated that IQOS is less harmful compared to cigarettes (43.8% and 38.1% among retailers from POS in Jewish and Arab neighborhoods, respectively).

Table 3 lists the retailers’ interactions with a PMI representative and PMI’s reaction to the POS display ban. More retailers from POS in Arab than Jewish neighborhoods reported having any form of interaction with a PMI representative (80.0% vs. 51.2%, *p=0.029*) with no statistically significant differences in regards to the detailed nature of those interactions.

PMI representatives assisted the majority of POS implement the display ban (89.5% in Arab and 77.3% in Jewish neighborhoods), the only borderline significant difference was more POS in Arab neighborhoods being advised on how to navigate and overcome regulatory restrictions, (26.3% vs. 6.8%, *p=0.05*) (Table 3). This included, for example, being given instructions on how to arrange the products behind the cover to make it easier to access and sell them, being directly informed about new campaigns and promotions, and repeatedly given information about the products.

### POS audits

#### POS characteristics

Table 4 summarizes the POS characteristics, marketing material, placement, promotion and regulatory compliance data from all audited POS, and across Arab vs. Jewish neighborhoods. The audited POS were mainly convenience stores not within a gas station (45.0%, n=58) or convenience stores within a gas station (31.0%, n=40). Significantly more POS in Arab neighborhoods were located in areas of low SES (75.0%, n=36), compared to only 2.4% (n=2) of POS in Jewish neighborhoods (*p<0.001*) (Table 4).

#### Marketing materials, prices and price promotions

**Figure 2:**
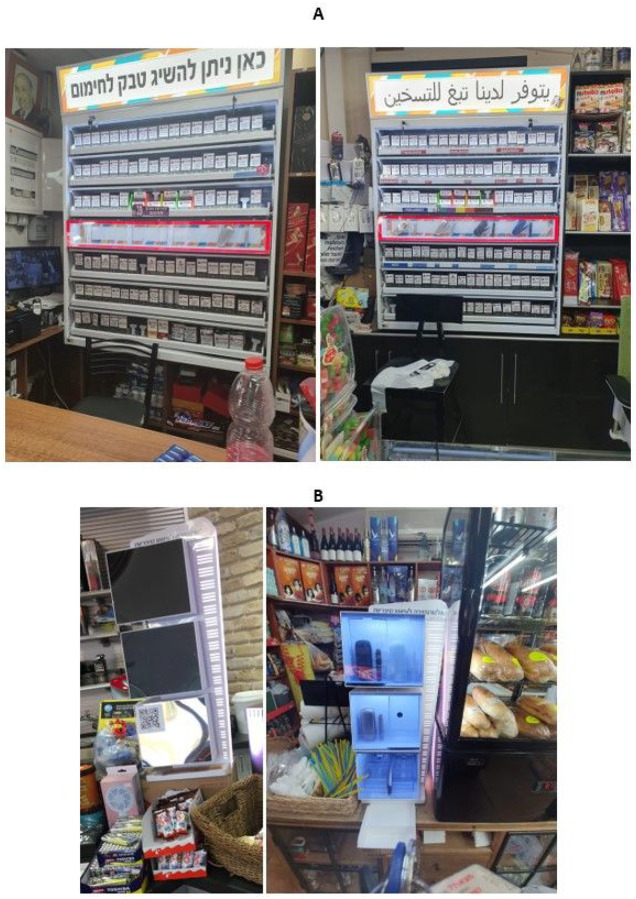
**A.**IQOS/HEETS special display cases nested within a display case for cigarettes: On the left: IQOS special display with the Hebrew words for “here you can buy heated tobacco”. On the right: IQOS special display with the Arabic words for “we have heated tobacco”. Both displays use IQOS’ brand colors, sell the IQOS device, are non-compliant with the display ban, have a special placement for HEETS in the colored section on top of the where the device is displayed with HEETS price stickers. The picture on the left also contains tobacco products not in plain packaging. Both are from POS in Arab neighborhoods. **B.** Special displays for only IQOS/HEETS: IQOS special display cases with the Hebrew words for “alternative for smoking cigarettes”. The one on the left is with the light turned off and has a QR code, and the one on the right is with the light turned on. Both are found at POS in Jewish neighborhoods.

The vast majority of POS (79.8%, n=103) had internal and/or external ads for any tobacco or nicotine product (72.9% Arab, n=35 and 82.9% Jewish, n=68); of which more than half was IQOS-indirect internal signage (57.1% Arab, n=20/35 and 57.4% Jewish, n=39/68), such as signs that said “heated tobacco units”, or “here you can buy heated tobacco”. The majority of POS that had any IQOS/HEETS signage included HEETS brand colors (70.0% Arab, n=14/20 and 79.5% Jewish, n=31/39), and IQOS/HEETS special displays (55.0% Arab, n=11/20 and 71.8% Jewish, n=28/39) (Table 4). Some of the special display cases provided by PMI were “discreet”; they had a light switch that makes the product visible only when turned on (figures 2A and 2B: IQOS special displays in Arab and Jewish neighborhoods, respectively).

The majority of POS in Arab (74.3%, n=26/35) and Jewish neighborhoods (85.3%, n=58/68) had cigarettes-specific internal signage, such as signs that said “cigarettes”. In contrast, significantly more POS in Arab neighborhoods mentioned a specific cigarette brand name (46.2%, n=12/26 vs. 22.4% in Jewish neighborhoods, n=13/58; *p=0.024*).

On average, POS in Arab neighborhoods carried fewer HEETS flavors (3.5 vs. 4.6, *p=0.001*), and sold them at a lower price (29.5 NIS vs. 30.7 NIS, *p<0.001*). Similarly, POS in Arab neighborhoods sold PMI cigarettes at a lower price on average (cheapest PMI cigarette: 24.5 NIS vs. 26.1 NIS, *p<0.001*; most expensive PMI cigarette: 38.4 NIS vs. 38.9 NIS, *p=0.004*). Price stickers that either indicated the advertised price or a price promotion were found at significantly more POS in Jewish neighborhoods (74.1%, n=43/58 vs. 65.4% in Arab neighborhoods, n=17/26; *p=0.044*).

#### Placement, visibility and regulatory compliance

IQOS/HEETS were placed within 1 m of the floor only in POS in Jewish neighborhoods (12.2%, n=10). IQOS was highly visible in the POS that sold it (68.8% in Jewish and 66.7% in Arab neighborhoods), but the visibility of HEETS was higher among POS in Jewish neighborhoods (62.2% vs. 39.6% in Arab neighborhoods, *p=0.010*).

A significantly higher proportion of POS in Arab neighborhoods had products in their original packaging (i.e., not in plain packaging as required by law) compared to Jewish neighborhoods’ POS (37.5% vs. 20.7%, *p=0.027*), but a higher proportion were compliant with the display ban (25.5% vs. 8.8%, *p=0.011*).

## Discussion

Findings from these studies show that, in general, PMI employed similar marketing strategies at POS in Arab and Jewish neighborhoods, without clear specific targeting. Nonetheless, a more personalized marketing approach (personal communication, social events) was more prevalent in POS in Arab neighborhoods.

Our findings of no over-targeting of POS in Arab neighborhoods compared to Jewish ones is in alignment with our previous study that explored PMI’s marketing in print media across population groups and media outlets in Israel.^[25, 26, 29]^ However, this is contradictory to research in other countries, where clear targeting of minority populations was reported.^[19–22]^ This might be attributed to IQOS being a relatively new product in the market, which would warrant a focus on the majority population in order to increase market share. However, it is surprising that despite PMI’s claims that this product is intended for adult smokers,^[26, 30]^ there seems to be no apparent targeting of Israel’s largest smoking population – the Arab population.^[26, 28]^

The use of different marketing strategies for POS in Arab or Jewish neighborhoods, such as more personal communication with retailers and invitation to social events could be attributed to a form of close network marketing that might be due to cultural differences. It has been suggested that in the Arab population, business relationships might be heavily influenced by personal ties.^[31]^ In addition, this might reflect the presence of different marketing teams (Arab and Jewish) to carry out in-person communications and outreach activities.

The majority of surveyed retailers had a positive attitude towards IQOS/HEETS. These perceptions are of great importance because they might influence how retailers communicate with customers and could be the result of PMI’s marketing efforts directed at the POS. While some retailers talked about themselves becoming salespeople or intermediaries who connected potential customers with a PMI representative, retailers in Arab neighborhoods were more inclined to use their personal experiences using IQOS to increase their credibility when promoting the product to customers. The majority of POS in Arab neighborhoods were small businesses (i.e., grocery stores; 71.4%), owned and operated by community members who can promote IQOS sales by influencing their customers through personal relationships, cultural cohesion, and embedded trust.^[31]^

Our findings suggest that the POS were used by PMI as a tool to directly market IQOS to consumers, by sending saleswomen to set up small IQOS stands inside the POS, and allowing the representative the freedom to talk to customers in order to promote the product, both of which were more prevalent at POS in Arab neighborhoods. The use of saleswomen is a newer form of what was known in the past as “cigarette girls”, which was previously used by PMI’s branch in Australia in 2000.^[32]^

PMI’s active involvement in helping retailers implement the display ban might have also contributed to the retailers’ positive perception of IQOS, especially with signage that serve as constant reminders of the product. Research from Scotland also highlighted the tobacco industry’s role in helping POS implement display bans and how to work around them.^[33]^ A survey by The Israel Democracy Institute in 2021 indicated that Arab residents expressed lower trust in the local authorities (32.0% vs. 62.0% of Jewish residents),^[34]^ which might create an opportunity for external entities to influence the retailers by offering guidance, such as advising on how to work around the display ban.

Internal IQOS signage at audited POS was found in more than half the POS in Arab and Jewish neighborhoods. However, these were mostly indirect and did not explicitly state the brand name, but used more general statements (such as “heated tobacco”). Nonetheless, a high proportion of the signs (76.3%) used specific colors that correspond to HEETS flavors which could be interpreted as branded advertisement, and therefore forbidden by law. Additionally, we found that some retailers were provided with display cases that only show the product when a switch is turned on, thereby subtly violating the display ban. These findings strengthen previous results showing the various ways in which PMI circumvents legislation.^[3, 35]^

## Limitations

These two studies used cross-sectional data that might not be reflective of all POS in Arab and Jewish neighborhoods in Israel. We only assessed POS in major cities, and our data collection efforts resulted in a small sample size for both studies. PMI might have employed other marketing tactics in other cities, or in the periphery. However, the IQOS website list of POS includes very few IQOS POS located in other dominant Arab cities and periphery localities. Additionally, we collected data both via phone, online, and face-to-face, with differences in data collection between Arab and Jewish POS (Jewish POS 79.2% by phone/online [n = 38/48], Arab 76.2% face-to-face [n = 16/21]), which might have impacted our results.

The majority of POS in Jewish neighborhoods were of middle- and high-SES, while the POS in Arab neighborhoods were of either medium- or low-SES, suggesting that differences might be based on economic, rather than ethnic factors, which cannot be differentiated within the scope of these studies. In addition, there might be some differences based on other factors, such as the city, including predominant population (mixed, predominant Arab, predominant Jewish), and store type. The small sample size precluded us from running more analyses to adjust for these factors. The data for both studies was collected at different points in time, which might have impacted the results. However, there were no differences in legislation or implementation during these times.

Nazareth was the only majority Arab city included in these studies, and it has both a Christian and Muslim population which could have affected the results. Currently, there is no available data on differences in smoking rates between Muslims and Christians, but a study conducted in 2012 showed that Muslim Arabs had a higher secondhand smoking exposure at home (55.4%), compared to 49.0% of Christian Arabs.^[36]^ This could be attributed to differences in smoking behaviors in these two subpopulations.

## Conclusions

Even though IQOS marketing was very similar across POS in Arab and Jewish neighborhoods, PMI utilized marketing elements of cultural significance, especially for POS in Arab neighborhoods, such as more personal communication and invitation to social events. Despite Israel having both a POS advertisement ban and a display ban in place, our results indicate a high level of regulatory non-compliance and legislation circumvention in both Arab and Jewish populations, stressing the need for better implementation and enforcement. Additional research with continuous surveillance is needed to fully understand demographic and ethnic-based differences in tobacco advertising and to reduce smoking-related health disparities.

## Figures and Tables

**Table 1.** POS and retailer characteristics, overall and across Arab vs. Jewish neighborhoods

Variable		Population group		p value^†^
	Total (n=69)	Arab (n=21)	Jewish (n=48)	
	n (%)	n (%)	n (%)	
***POS characteristics***				
**Store Type**				
Convenience store without gas	24 (34.8)	**4 (19.0)**	**20 (41.7)**	0.001^#^
Convenience store with gas	8 (11.6)	**0 (0.0)**	**8 (16.7)**	
Grocery store	26 (37.7)	**15 (71.4)**	**11 (22.9)**	
Liquor store	5 (7.2)	**0 (0.0)**	**5 (10.4)**	
Tobacco store	4 (5.8)	**1 (4.8)**	**3 (6.2)**	
Other (café, kiosk)	2 (2.9)	**1 (4.8)**	**1 (2.1)**	
**Neighborhood socio-economic status (SES)**				
Non-residential	7 (10.1)	**0 (0.0)**	**7 (14.6)**	0.009^#^
Low	10 (14.5)	**5 (23.8)**	**5 (10.4)**	
Medium	42 (60.9)	**16 (76.2)**	**26 (54.2)**	
High	10 (14.5)	**0 (0.0)**	**10 (20.8)**	
***Participants characteristics***				
**Age, mean (SD)**	39.1 (9.9)	39.4 (9.5)	38.9 (10.1)	0.806^
**Sex**				
Male	62 (89.9)	17 (81.0)	45 (93.8)	0.188^#^
Female	7 (10.1)	4 (19.0)	3 (6.2)	
**Position at the POS**				
Owner	16 (23.2)	4 (19.0)	12 (25.0)	0.760^#^
Manager	53 (76.8)	17 (81.0)	36 (75.0)	
**Cigarette smoking status** *				
Current smoker	26 (38.2)	7 (33.3)	19 (40.4)	0.833
Past smoker	14 (20.6)	5 (23.8)	9 (19.2)	
Never smoker	28 (41.2)	9 (42.9)	19 (40.4)	
**IQOS use status** *				
Current user	8 (11.8)	1 (4.8)	7 (14.9)	0.163^#^
Past user	9 (13.2)	5 (23.8)	4 (8.5)	
Never user	51 (75.0)	15 (71.4)	36 (76.6)	

POS – Point-of-sale.

† Chi square test, unless stated otherwise.

# Fishers exact test.

^ Mann-Whitney test.

* Missing: **Participants characteristics:** Cigarette smoking n=1; IQOS use status n=1 (both are retailers in Jewish neighborhoods). **Bold** indicates between-group statistically significant differences (Bonferroni correction).

**Table 2.** POS IQOS/HEETS marketing strategies, overall and across Arab vs. Jewish neighborhoods

Variable		Population group		p value^†^
	Total (n=69)	Arab (n=21)	Jewish (n=48)	
	n (%)	n (%)	n (%)	
**Number of HEETS flavors** *				
<4	22 (32.8)	**14 (66.7)**	**8 (17.4)**	<0.001
4 – 5	20 (29.9)	5 (23.8)	15 (32.6)	
All 6	25 (37.3)	2 (9.5)	23 (50.0)	
**Special display** *	30 (44.8)	5 (25.0)	25 (53.2)	0.034
**Promotions to POS** *				
Free HEETS samples	13 (21.3)	4 (20.0)	9 (22.0)	1.000
Price discounts for the retailer’s own purchase of HEETS/IQOS	18 (29.5)	8 (42.1)	10 (23.8)	0.174
Paraphernalia	7 (10.6)	6 (30.0)	1 (2.2)	0.002^#^
Other gifts^§^	6 (9.7)	4 (20.0)	2 (4.8)	0.079^#^
Price discounts, rebates, or incentives based on promoting their products	27 (41.5)	7 (36.8)	20 (43.5)	0.621
Incentives for sales of their products	22 (33.8)	5 (25.0)	17 (37.8)	0.315
Invitations to IQOS parties or events	20 (29.9)	10 (47.6)	10 (21.7)	0.032
**Promotions to customers** *				
Free HEETS samples	4 (6.7)	1 (6.3)	3 (6.8)	1.000^#^
Other gifts^‡^	5 (8.3)	3 (18.8)	2 (4.5)	0.112^#^
Price promotions^$^	7 (11.9)	1 (7.1)	6 (13.3)	1.000^#^
Price discounts	23 (37.7)	3 (18.8)	20 (44.4)	0.069
Coupons	6 (10.5)	1 (6.7)	5 (11.9)	1.000^#^
Special prices for members	4 (6.9)	2 (12.5)	2 (4.8)	0.303^#^
**Advertisements by POS retailer** *				
Any form of ads	38 (58.5)	10 (50.0)	28 (62.2)	0.356
Online	5 (7.7)	0 (0.0)	5 (11.1)	0.313^#^
Social media	3 (4.8)	1 (5.0)	2 (4.7)	1.000^#^
Print media	2 (3.1)	1 (5.0)	1 (2.3)	0.531^#^
Inside the POS	37 (58.7)	10 (50.0)	27 (62.8)	0.337

POS – Point-of-sale.

† Chi square test, unless stated otherwise.

# Fishers exact test.

* Missing: **Number of HEETS flavors** n=2; **Special display** n=2; **Promotions to POS**: free HEETS samples n=8; price discounts for your own purchases n=8; paraphernalia n=3; other gifts n=7; price discounts, rebates, or incentives based on promoting their products n=4; incentives for sales n=4; invitations to parties n=2; **Promotions to costumers:** free HEETS samples n=9; paraphernalia is zero for all POS; other gifts n=9; price promotions n=10; price discounts n=8; coupons n=12; special prices for members n=11; Special discounts for military/students was zero for all POS; **Advertisements**: any form of ad n=4 (if all items were missing); online n=4; social media n=6; print media n=5; inside the POS n=6.

§ Other gifts given to retailers included lighters, lighter stands swimming competitions, and flights abroad.

‡ Other gifts given to customers included lighters.

$ Price promotions offered to customers such as buy one get one free, 2 NIS off the price of HEETS, or buy IQOS and get a free HEETS package. **Bold** indicates between-group statistically significant differences (Bonferroni correction).

**Table 3.** Interactions with a PMI representative and PMI’s reaction to the POS display ban, overall and across POS in Arab vs. Jewish neighborhoods

Variable		Population group		p value^†^
	Total (n=69)	Arab (n=21)	Jewish (n=48)	
	n (%)	n (%)	n (%)	
**Specific IQOS/HEETS salesperson** *	25 (37.9)	6 (28.6)	19 (42.2)	0.287
**Interaction with PMI salesperson** *				
Any interaction	38 (60.3)	16 (80.0)	22 (51.2)	0.029
Provided direction on placement	32 (50.0)	13 (65.0)	19 (43.2)	0.106
Provided information on the target market	16 (25.8)	2 (10.0)	14 (33.3)	0.050
Provided direction how to communicate with consumers	21 (35.0)	7 (35.0)	14 (35.0)	1.000
Provided information on IQOS/HEETS vs. other tobacco products	31 (51.7)	13 (65.0)	18 (45.0)	0.144
**PMI’s reaction to the POS display ban**				
Any interference*	51 (81.0)	17 (89.5)	34 (77.3)	0.318
Provided education regarding the tobacco legislation	26 (37.7)	11 (57.9)	15 (34.1)	0.096
Advised on how to work around the tobacco legislation	8 (11.6)	5 (26.3)	3 (6.8)	0.050^#^
Provided free cabinets, display cases and/or signage to address the tobacco legislation	36 (52.2)	12 (63.2)	24 (54.5)	0.585
Changed their promotional strategies for products such as e-cigarettes and/or IQOS/HEETS	7 (10.1)	3 (15.8)	4 (9.1)	0.667^#^
Minimized the importance of compliance with the tobacco legislation	4 (5.8)	1 (5.3)	3 (6.8)	1.000^#^
Sold cabinets, display cases and/or signage to address the tobacco legislation	8 (11.6)	0 (0.0)	8 (18.2)	0.095^#^

POS – Point-of-Sale. PMI – Philip Morris International.

$ Includes ticking at least one statement from a-g.

† Chi square test, unless stated otherwise

# Fisher’s exact test.

* Missing: Specific IQOS/HEETS salesperson n=3; **Interaction with a PMI salesperson:** Any interaction n=6; provide direction on placement n=5; target market n=7; communicate with consumers n=9; information on product n=9; **PMI’s reaction to the POS display ban:** All items n=6.

**Table 4.** POS characteristics, marketing material, placement, promotions and regulatory compliance, overall and across Arab vs. Jewish neighborhoods

Variable	Total (n=129) n (%)	Population group		p value^†^
		Arab (n=48)	Jewish (n=81)	
		n (%)	n (%)	
**POS characteristics**				
*Type of store*				
Convenience store with gas	40 (31.0)	11 (22.9)	29 (35.8)	0.440
Convenience store without gas	58 (45.0)	23 (47.9)	35 (43.2)	
Grocery store/ supermarket	21 (16.2)	9 (18.8)	12 (14.8)	
Other^Ÿ^	10 (7.8)	5 (10.4)	5 (6.2)	
*Neighborhood socioeconomic status (SES)*				<0.001
Non-residential	17 (13.2)	4 (8.3)	13 (15.9)	
Low	37 (28.7)	**36 (75.0)**	**2 (2.4)**	
Medium	56 (43.4)	**7 (14.6)**	**49 (59.8)**	
High	19 (14.7)	**1 (2.1)**	**18 (21.9)**	
**Marketing materials (any)**	103 (79.8)	35 (72.9)	68 (82.9)	0.131
***IQOS/HEETS***				
Any ad/sign^¶^	59 (57.3)	20 (57.1)	39 (57.4)	0.475
Special display^‡^	39 (66.1)	11 (55.0)	28 (71.8)	0.142
Brand colors^‡^	45 (76.3)	14 (70.0)	31 (79.5)	0.111
***Cigarettes***				
Any ad/sign^¶^	84 (81.6)	26 (74.3)	58 (85.3)	0.162
Brand names*	25 (29.8)	12 (46.2)	13 (22.4)	0.024
Price stickers*	60 (71.4)	17 (65.4)	43 (74.1)	0.044
**Visibility** **				
IQOS	16 (76.2)	4 (66.7)	11 (68.8)	1.000^#^
HEETS	70 (54.3)	19 (39.6)	51 (62.2)	0.010
Cigarettes	81 (62.8)	27 (56.3)	54 (65.9)	0.237
**HEETS flavors; M (SD)**	4.2 (1.8)	3.5 (1.9)	4.6 (1.6)	0.001^
**IQOS/HEETS Placement**				
Within 30 cm of toys or candy	20 (15.5)	5 (10.4)	15 (18.3)	0.219
Within 1 m of the floor	10 (7.8)	0 (0.0)	10 (12.2)	0.013^#^
**Price promotion**				
IQOS/HEETS	14 (10.9)	4 (8.3)	10 (12.2)	0.479
Other tobacco product	33 (25.6)	9 (18.8)	24 (29.3)	0.171
**Prices; M (SD)**				
HEETS	30.2 (1.7)	29.5 (1.2)	30.7 (1.9)	<0.001^
Cheapest PMI cigarette	25.4 (2.1)	24.5 (1.5)	26.1 (2.3)	<0.001^
Most expensive PMI cigarette	38.5 (3.2)	38.4 (1.4)	38.9 (4.0)	0.004 ^
**Regulatory compliance**				
Minimum age signage	75 (58.1)	23 (47.9)	52 (63.4)	0.070
No smoking sign	37 (28.7)	18 (37.5)	19 (23.2)	0.088
Plain packaging	95 (73.6)	30 (62.5)	65 (79.3)	0.027
Display ban^&^	19 (15.1)	12 (25.5)	7 (8.8)	0.011

POS – Point-of-Sale.

† Chi square test, unless stated otherwise.

# Fisher’s exact test.

^ Mann-Whitney test.

Ÿ Other: Liquor store n=1; Tobacco shop n=3; Coffee shop n=3; Candy store n=2; Spice shop n=1.

¶ Out of those with any internal ad (n=103; Arab n=35 and Jewish n=68).

‡ Out of those with any IQOS ads (n=59; Arab n=20 and Jewish n=39).

* Out of those with any *cigarette* ads (n=84; Arab n=26 and Jewish n=58).

** Not sold: IQOS device n=108 (n=42 Arab and n=66 Jewish).

& Excluding n=3 tobacco shops (Arab n=1 and Jewish n=2) that the display ban does not apply to. **Bold** indicates between-group statistically significant differences (Bonferroni correction).

## Data Availability

The datasets used and/or analyzed during the current study are available from the corresponding author on reasonable request.

## Abbreviations

HTP: Heated tobacco product
NIS: New Israeli Shekel
PM: Philip Morris
PMI: Philip Morris International
POS: Point-of-sale
RA: Research assistant
SD: Standard deviation
SES: Socioeconomic status
STARS: Standardized Tobacco Assessment for Retail Settings
US: United States
